# The impact of natural modes in plasmonic imaging

**DOI:** 10.1038/srep18247

**Published:** 2015-12-16

**Authors:** Angela Demetriadou

**Affiliations:** 1 Department of Chemistry, Imperial College London, London, SW7 2AZ, United Kingdom; 2 Department of Physics, Imperial College London, London, SW7 2AZ, United Kingdom

## Abstract

Plasmonic imaging is crucial for understanding cellular behaviours for biological sciences, where is used to image and track organelles in cells, such as DNA and virus molecules. Due to the fast dynamics of the intra-cellular processes, it is essential to keep the cells under their native states (i.e. label-free), establishing plasmonic imaging as one of the most powerful tools for studying biological samples. In this article, a theoretical model is presented that accurately predicts the properties of a plasmonic image, paving the route towards the characterization of an imaged nano-object. It is shown that natural modes are not only excited, but actually dominate the intensity and shape of the observed plasmonic image. Hence, the proposed model explains the dynamics forming the plasmonic image and can be used to extract spectroscopy information from current plasmonic imaging techniques.

Surface Plasmon Polaritons (SPPs) are bound electromagnetic waves on metal-dielectric interfaces. The evanescent nature of SPP-waves has led to broad applications for sensing purposes^1^,^2^,^3^,^4^,^5^, imaging interfacial structures^6^ and ultrathin organic and bipolymer films^7^,^8^, enabled by the extreme field enhancement at the metal-dielectric interface that accompanies SPP-waves. In biological sciences, plasmonic imaging (i.e. imaging with SPP-waves) is commonly used to track and image nano-sized organelles in cells with nanometre precision^9^, where a label-free and real-time imaging technique is crucial to map and understand the fast intra-cellular dynamics at their native state. In fact, this technique has already been used for label-free imaging of single DNA^10^ and virus^11^ molecules, tracking mitochondria transportation^9^ and *in-situ* imaging of particle adsorption on a thin gold film^12^. It overcomes common problems of fluorescence microscopy, which always requires labelling the sample that ‘disturbs the native dynamic processes of under study’^9^. For example, the label agents (i.e. fluorophore molecules) can elongate and twist the native nature of a DNA molecule and affect its charge properties^13^,^14^. Also, fluorophore molecules have a weak fluorescence emission, requiring long acquisition times and therefore limit the imaging speed and fluorescence microscopy to record only slow cellular processes. Additionally, plasmonic imaging has been widely used in to map electro-catalytic activity of single nanoparticles, “enabling voltammetry and amperometry with high spatial resolution and sensitivity”^15^,^16^. Hence, plasmonic imaging is crucial for biological and electro-catalytic sciences and therefore is vital to understand the electromagnetic interaction forming the image, and extract further information about the object from its plasmonic image.

The properties of the plasmonic image change for nanoparticles of different sizes and composition. The plasmonic image properties can be explained using a full-wave analytical model^17^ that maps the electromagnetic interaction behind the image’s formation. This theory models the SPP-wave diffraction from a spherical particle, and is applicable from sub-wavelength to macroscopic nanoparticles^17^. Therefore, it goes beyond the electrostatic approach of a dipolar approximation (that is limited to nanoparticles much smaller than the wavelength of light^18^,^19^,^20^), the implementation of Green’s functions^21^, (which require a semi-analytical solution for particles away from the quasi-static limit) and the use of effective boundary conditions^22^ (that require a full-numerical solution of integral equation^23^,^24^,^25^). The analytical model^17^ can also be easily extended for THz SPPs, which have been recently exploited for time-resolved imaging of *μm*-particles^26^. Most importantly though, the shape, dimensions and intensity of the plasmonic image are accurately predicted. However, it would be desirable to obtain information about the object by simply observing the plasmonic image (i.e. enabling spectroscopic characterization in addition to imaging and tracking of the object).

In this article, it is explained how the plasmonic images’ properties change with the nanoparticles’ composition and size, and illustrative maps for the most common cases are provided and discussed. Initially, a brief description is given for the analytical model^17^ describing the diffraction of a SPP-wave (i.e. an evanescent wave) by an object, on which the derivation of the ‘SPP-scattering and extinction cross-sections’ is based. The advantage of the analytical model to calculate a plasmonic image in seconds and an SPP-cross-section line in tens of seconds, while numerical calculations require days for accurate results (see Methods), provides great flexibility to obtain information for plasmonic imaging. The definition of ‘SPP-cross-sections’ was chosen due to the fact that they measure the diffraction under SPP-excitation (‘SPP’ accounts for the non-uniform character of the SPP-excitation following the exponential evanescent decay of the SPP). It should be emphasized that the SPP-cross-sectional quantities are by definition different to the scattering and extinction cross-sections derived in Mie theory, since Mie theory deals only with plane wave diffraction by a scatterer and not a SPP-wave. The SPP-scattering and extinction cross-sections enable spectroscopic information to be extracted from the plasmonic image, expanding the capabilities of plasmonic imaging techniques. Despite the exponential decay of the SPP-wave excitation, which limit it to interact with part of the nanoparticle, natural modes are excited, and in fact govern the properties of the plasmonic image.

## Results

### Plasmonic image of an isolated nanoparticle

**Figure 1:**
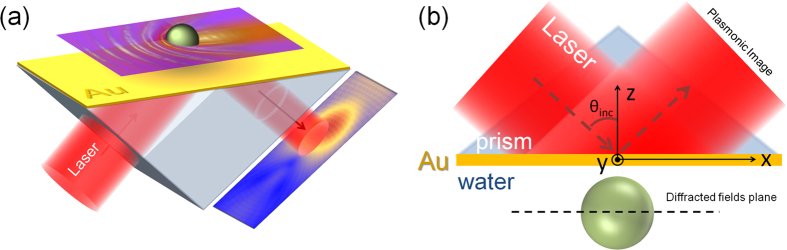
The Kretschmann configuration set-up (**a**) illustrating the plasmonic image formation and (**b**) the axes of the system used for the analytical model.

The evanescent nature of SPP-waves allows for massive field enhancement at the metal-dielectric interface, which exponentially decays away from it. However, this also means that SPP-waves can only be excited under certain conditions and set-ups. In this article, I choose to consider the Kretschmann configuration (see Fig. 1), since it is among the easiest and most popular set-ups, although our findings are applicable for any SPP-excitation set-up. The Kretschmann configuration is consisted of a prism with refractive index *n*_1_ and a thin layer of metal with thickness *d*_*m*_ and refractive index *n*_2_, deposited on one of the prism’s sides. The metal slab is illuminated with a laser through the prism, with electromagnetic fields **H** = (*H*_*x*_, *H*_*y*_, *H*_*z*_) and **E** = (*E*_*x*_, *E*_*y*_, *E*_*z*_), guided by **k**_**0**_ = (*k*_*x*_, *k*_*y*_, *k*_*z*_) (the system’s axes are defined in Fig. 1) and excite an SPP-wave in medium 3 when *n*_3_ < *n*_1_. Only the TM mode of the incident wave (i.e. *H*_*z*_ = 0 component) can couple evanescently to a SPP^27^. Assuming that the nanoparticles interacting with the SPP-wave are spherical, then the field equation describing the excited SPP wave in medium *n*_3_ needs to be written in spherical harmonics, and follows from Maxwell’s equations^17^:


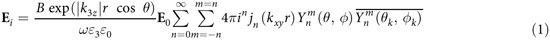


where *k*_*x*_, *k*_*y*_, 
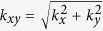
 and *k*_3*z*_ are the SPP wavevectors in medium *n*_3_, given by *k*_*x*_ = *n*_1_|*k*_0_| sin *θ*_*inc*_ cos *ϕ*_*inc*_, *k*_*y*_ = *n*_1_|*k*_0_| sin *θ*_*inc*_ sin *ϕ*_*inc*_ and 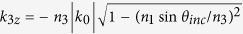, where *θ*_*inc*_ and *ϕ*_*inc*_ are the laser’s angles of incidence on the metal slab, *j*_*n*_ is the spherical Bessel function, *r*, *θ* and *ϕ* are the spatial spherical coordinates with origin at a random point (*x*_0_, *y*_0_, *z*_0_), (defined as 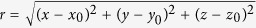, 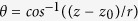 and 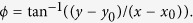

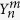
 is the spherical harmonic function (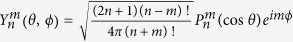 where 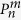 is the associated Legendre Polynomial), 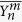 is its complex conjugate and *B* is a coefficient dependent on the materials of the different regions/media and is the only term that needs to change for other SPP-excitations set-ups^17^. Since throughout this paper the sensor and incident radiation are kept the same, *B* remains a constant. The first term in equation (1) expresses the evanescent exponential decay of the SPP-wave’s amplitude away from the metal-dielectric interface, while the summation describes the phase propagation and **E**_0_ defines the vector direction of the SPP-wave^17^.

A spherical nanoparticle placed in medium 3, diffracts the excited SPP-wave into three channels^17^,^21^,^28^,^29^: (i) SPP-channel, where the scattered field retains its evanescent nature remaining bound on the metal-dielectric interface^17^:


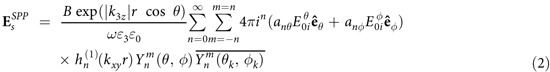


(ii) the radiative channel, where part of the incident SPP-wave decouples from the metal-dielectric interface and propagates (or radiates) away from the particle in medium 3^17^:


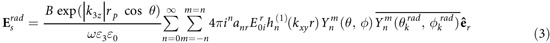


and (iii) the finite-slab (FS)-channel 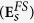, where the radiative component interacts with the finite metal slab. In the above equations, 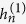 is the spherical Hankel function of the first kind, and *a*_*n*(*r*,*θ*,*φ*)_ are the scattering coefficients given by^17^:


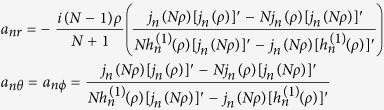


where *ρ* = *k*_*xy*_*r*_*p*_ is the size parameter.

Since the plasmonic image is always observed at exactly the reflection angle −*θ*_*inc*_ (i.e. it travels with the reflected beam from the metal slab), only the scattering into the SPP-channel carries the image’s phase information to the far-field, while the partially-reflected beam from the metal slab just adds a constant background, which here is neglected. The scattered fields into the SPP-channel are still bound on the metal-dielectric interface, propagating for several wavelengths. This causes local charge fluctuations on the metal-medium 3 interface, which are mirrored on the metal-prism interface (in a similar but reverse way to the initial excitation of the SPP-wave). These localized charge accumulations provide a resistance to the continuous excitation of the SPP-wave by the incident laser beam, leading to local enhancement to the reflected beam’s intensity, i.e. the plasmonics image. The radiative component 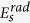 and 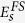 negligibly contribute to the formation of the plasmonic image since they are not restricted to propagate only at **k**_*refl*_ (i.e. they emit at all angles and not just at −*θ*_*inc*_). The contribution of 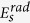 is negligibly small for dielectric nanoparticles, especially of sub-wavelength dimensions, but becomes stronger for metal nanoparticles. 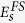 is even less significant, since it is the secondary diffraction of 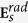 by the finite slab. Therefore, the plasmonic image for sub-wavelength nanoparticles can be expressed by:


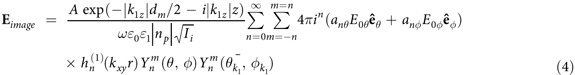


where 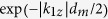 defines the amplitude of the decoupled wave that propagates to the far-field, *a*_*nθ*_, *a*_*nϕ*_, *E*_0*θ*_ and *E*_0*ϕ*_ define the shape of the plasmonic image and *I*_*i*_ is the intensity of the SPP wave incident on the nanoparticle that decays exponentially across the nanoparticle’s diameter, which is dependent on the radius of the nanoparticle *r*_*p*_ and its distnace *d*_*sep*_ from the metal slab, given by: 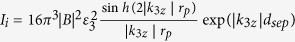. It follows from the above description and the fact that scattered fields in the SPP-channel propagate for several wavelengths on the metal-dielectric interface, that the plasmonic image of a nanometer-sized particle has *μm*-size dimensions.

**Figure 2:**
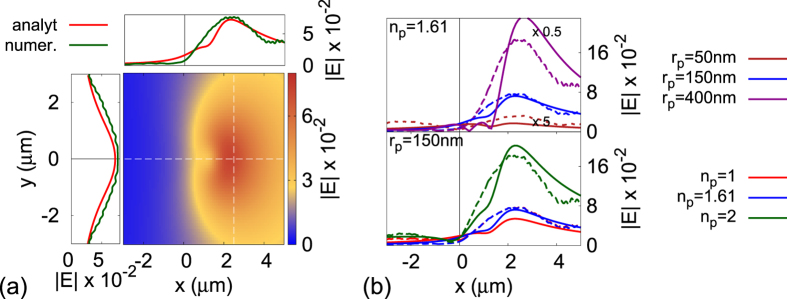
The plasmonic image for various nanoparticles. (**a**) The plasmonic image of a polystyrene (*n*_*p*_ = 1.61) nanoparticle of radius *r*_*p*_ = 150 *nm*). Analytical predictions are plotted with red full lines and numerical calculations with green full lines at the image’s centre. (**b**) The |*E*|-field profile of the plasmonic image of polystyrene nanoparticles of various sizes (top figure) and of nanoparticle of radius 150 *nm* and various refractive indices. Analytical predictions are plotted with full lines and numerical calculations with dashed lines.

In Fig. 2(a), the plasmonic image is plotted of a polystyrene nanoparticle (*n*_*p*_ = 1.61) of radius *r*_*p*_ = 150 *nm* for a Kretschmann configuration composed by a prism of *n*_1_ = 1.725, and a gold^30^ film of thickness *d*_*m*_ = 47 *nm* deposited on one side of the prism. A flow cell is attached on the other side of the metal slab, where an aqueous (i.e. *n*_3_ = 1.333) solution is assumed. A monochromatic wave (laser) of *λ*_0_ = 675 *nm* is illuminating at an angle *θ*_*inc*_ = 55.8° and *ϕ*_*inc*_ = 0 the gold film through the prism, exciting a SPP-wave on the water-gold interface of wavelength 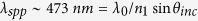. The plasmonic images predicted analytically (red lines) using (4) and calculated numerically (green lines) are plotted in Fig. 2(a) for a polystyrene nanoparticle (*n*_*p*_ = 1.61) of *r*_*p*_ = 150 *nm*. They show very good agreement, while as discussed above the image is of *μm* dimensions. Figure 2(b,c) show the intensity of the plasmonic image of dielectric nanoparticles along the x-axis for various sizes and refractive indices, calculated analytically (full lines) and numerically (dashed lines) at a distance of 950 *nm* away from the metal slab using the commercial software Finite-Difference Time-Domain (FDTD) solutions from Lumerical Solutions, Inc^31^. It is noteworthy that the full-width half-maximum (FWHM) of the plasmonic image does not change with the size of the sub-wavelength nanoparticles (i.e. *λ*_*SPP*_ ~ 473 *nm* > 2*r*_*p*_). Hence for nanoparticles of *r*_*p*_ < 200 *nm*, only the maximum intensity of the plasmonic image changes by varying the nanoparticle’s size. For nanoparticles larger than 400 *nm*, the SPP-wave is able to resolve the geometric details of the nanoparticle, since they are now comparable or larger than *λ*_*SPP*_. For nanoparticles of the same size and of different dielectric composition, the plasmonic image’s intensity also varies as shown in Fig. 2. The maximum intensity of the plasmonic image is clearly also dependent on the difference between the dielectric constants of the nanoparticle and its aqueous environment (*n*_3_ = 1.333). The plasmonic images in Fig. 2 obtained numerically and analytically are in very good agreement with experimental findings for silica nanoparticles (*n*_*p*_ ~ 1.45), H1N1 influenza virus A and cell organelles such as mitochondria, previously reported in the literature^9^,^11^. The theoretical model accurately predicts the sharp increase on the image’s intensity at *x* ~ 0 and its slow decay away from the peak. Experimental measurements for plasmonic images quite commonly show weak resonances on the intensity of the plasmonic image, which are most likely due to aberrations of the experimental set-up, since they are never seen either numerically or analytically.

### SPP-diffraction cross-sections

The diffracted fields expressed by (2) and (3) and the plasmonic image in (4) provide a valuable insight to the electrodynamics driving the formation of the nanoparticle’s image. However, to characterize the nanoparticle experimentally, one needs to quantify the properties of the plasmonic image. Since the full-width half-maximum of the plasmonic image remains constant for sub-wavelength nanoparticle, then its maximum intensity is usually recorded in the far-field experimentally^5^,^9^,^10^,^12^, providing an easy and direct way to obtain information about the imaged object. To relate the analytical model to the maximum intensity of the plasmonic image, and hence enable the characterization of the nanoparticle, I derive the scattering (*σ*_*scat*_) and extinction (*σ*_*ext*_) cross-sections, since they quantify the field disturbances caused by the diffraction. It should be emphasized that these cross-sectional characteristics are specifically applicable for a SPP-wave system and distinctively different to the classical cross-sections of a plane wave incidence described by Mie theory^32^, hence named ‘SPP-cross-sections’. The SPP scattering and extinction efficiencies 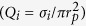 (derivation shown in Suppl. Materials) are given by:


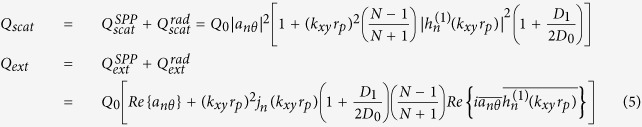


where 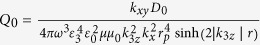, 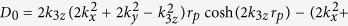 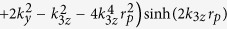, 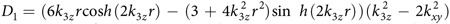 and 
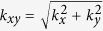
, *N* = *n*_*p*_/*n*_3_, with the absorption efficiency *Q*_*abs*_ given by: *Q*_*abs*_ = *Q*_*ext*_ − *Q*_*scat*_. It should be noted that  in  (5), the first term in the square brackets is due to the scattering and extinction in the SPP-channel and the second term is due to the radiative component.

**Figure 3:**
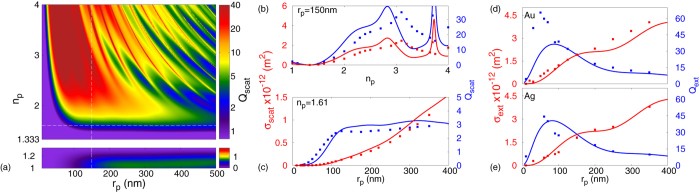
Scattering/Extinction cross-sections (*σ*_*scat*,*ext*_) and efficiencies (*Q*_*scat*,*ext*_) for various nanoparticles. (**a**) A scattering/extinction cross-section efficiency map for loss-less dielectric nanoparticles of various radii and refractive indices. (**b**) Polystyrene (*n*_*p*_ = 1.61) nanoparticles of varying radius, (**c**) of radius *r*_*p*_ = 150 *nm* and varying dielectric constant, (**d**) gold (Au) and (**e**) silver (Ag) nanoparticles of varyingradius. Analytical predictions are plotted with full lines and numerical calculations with points.

In Fig. 3, *σ*_*ext*_ and *σ*_*scat*_ are plotted for both dielectric and metallic nanoparticles. For loss-less dielectric nanoparticles (i.e *σ*_*ext*_ = *σ*_*scat*_), Fig. 3(a–c) show *σ*_*scat*_ and *Q*_*scat*_ for varying sizes of nanoparticle and refractive indices. As it is expected *σ*_*scat*_ increases for larger nanoparticles and for larger values of the refractive index. More specifically, in Fig. 3(b) *σ*_*scat*_ and *Q*_*scat*_ are plotted for nanoparticles of radius *r*_*p*_ = 150 *nm* made of different loss-less dielectric materials. When the nanoparticle has a refractive index identical to its surrounding medium (*n*_*p*_ = *n*_3_ = 1.333 ⇒ *N* = *n*_*p*_/*n*_3_ = 1), *σ*_*scat*_ and *Q*_*scat*_ take zero values as expected. For nanoparticles with refractive index smaller than the surrounding medium’s (*N* < 1), *σ*_*scat*_ and *Q*_*scat*_ increase with decreasing *N*, since the difference to the two refractive indices cause the SPP-wave to diffract more. As *N* becomes larger than unity, one can see that both *σ*_*scat*_ and *Q*_*scat*_ increase more rapidly. In fact at *n*_*p*_ ~ 3.1 they reach a maximum value. At this point, the wavelength of the SPP inside the nanoparticle is comparable to the particle’s radius (*λ*_*SPP*_/*n*_*p*_) ~ *r*_*p*_, and therefore the induced displacement current inside the dielectric particle is allowed to complete a loop, exciting an ‘SPP-induced natural mode’ (discussed in section 5 - again the term ‘SPP’ accounts for the non-uniform character for the modes excitation following the exponential decay of the SPP-wave). The analytical model accurately predicts all the *Q*_*scat*_ and *σ*_*scat*_ features observed numerically, despite the very high values of *n*_*p*_.

Figure 3(c) shows *σ*_*scat*_ and *Q*_*scat*_ behaviour for polystyrene nanoparticles (i.e. *n*_*p*_ = 1.61) of varying sizes. *σ*_*scat*_ always increases with the size of the nanoparticle, but the scattering efficiency *Q*_*scat*_ that is normalized to 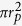, behaves differently. One can easily distinguish from *Q*_*scat*_ the sub-wavelength, meso-scopic and macroscopic regions (with respect to *λ*_*SPP*_ ~ 473 *nm*). For the sub-wavelength region (2*r*_*p*_ ≪ *λ*_*SPP*_/2 ⇒ *r*_*p*_ ≤ 110 *nm*), the *Q*_*scat*_ increases exponentially with *r*_*p*_, since as the nanoparticle’s size increases and diffracts the SPP-wave more strongly. For meso-scopic and larger nanoparticles (2*r*_*p*_ ~ *λ*_*SPP*_), where the nanoparticle’s size is comparable or larger than *λ*_*SPP*_, *Q*_*scat*_ reaches a plateau. For these nanoparticle sizes, the amplitude of the incident SPP field varies significantly across the nanoparticle, since the SPP decays exponentially away from the metal slab (for these calculations the evanenscent decay length of the SPP-wave is (1/|*k*_3*z*_|) ~ 144 *nm*). Hence, as *r*_*p*_ values reach the meso-scopic regime, the SPP-wave’s amplitude incident on the nanoparticle varies massively across the nanoparticle, with the south pole (Fig. 1(b)) of the particle (i.e. the outer most point with respect to the Au film) interacting with a weaker field amplitude than its north pole (Fig. 1(b)), leading to a plateau for *Q*_*scat*_. The FDTD numerical calculations (points) are in excellent agreement with our analytical predictions (lines) for both *σ*_*scat*_ and *Q*_*scat*_. Actually the analytical model predicts all the features observed numerically for both the scattering cross-section and efficiency. Hence, the analytical model is used to calculate a *Q*_*scat*_-map for loss-less dielectric nanoparticles, plotted in Fig. 3(a), where higher order natural modes can be seen. Additionally, the extinction cross-section and efficiency for gold and silver nanoparticles is plotted in Fig. 3(d–e), where again a good agreement is observed. The disagreements for the smallest nanoparticles are due to inaccuracies in our numerical calculations caused by computational power limitations, since a *μm*-sized image needs to be recorded, while sufficiently resolving a *nm*-sized nanoparticle. These inaccuracies are obviously more prominent for metal than dielectric nanoparticles, where the refractive index difference between the nanoparticle and its environment is more significant, demanding higher calculation resolutions and therefore computational power.

## Discussion

### Plasmonic image

**Figure 4:**
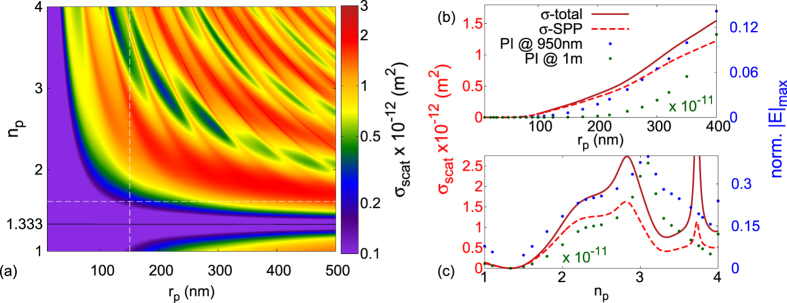
Scattering cross-section (*σ*_*scat*_) relation to the plasmonic image. (**a**) *σ*_*scat*_ map for loss-less dielectric nanoparticles. *σ*_*scat*_ (solid line), 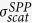 (dashed line) and the plasmonic images’ maximum |*E*|-field (points) dependence on (**b**) *r*_*p*_ and (**c**) *n*_*p*_ for loss-less dielectric nanoparticles. The maximum |*E*|-field values are numerically recorded at 950 *nm* (blue points) and 1*m* (green points) above the metal slab of the Kretschmann configuration.

**Figure 5:**
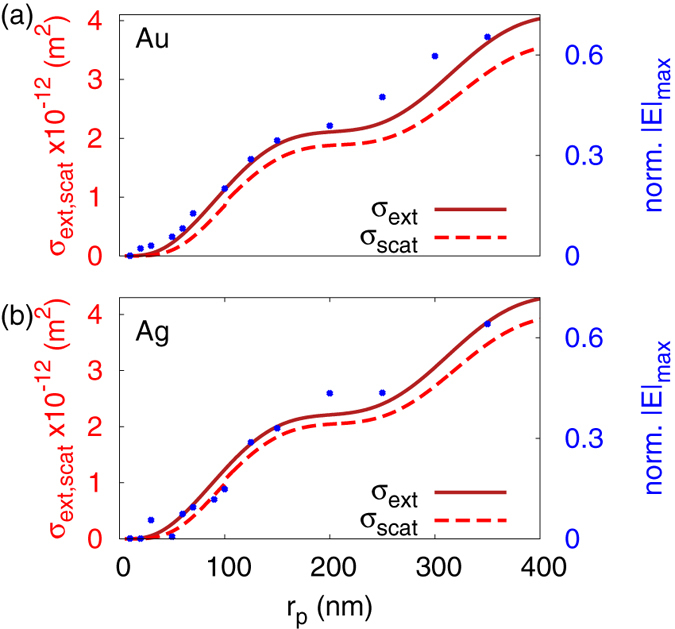
The extinction (*σ*_*ext*_ in solid lines) and scattering (*σ*_*scat*_ in dashed lines) cross-sections for (**a**) gold and (**b**) silver nanoparticles calculated analytically (lines) and plotted with the maximum value of their plasmonic image calculated numerically (points) at 560 *nm* away from the metal slab.

As discussed above and in Demetriadou A. *et al.*^17^, the plasmonic image is created due to the diffracted fields of the SPP-wave, inducing charge fluctuations at the metal-dielectric interface. The SPP-extinction and scattering cross-sections quantify this process and in Fig. 4(b,c), 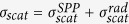 and 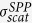 are plotted for loss-less dielectric nanoparticles along with the maximum value of their corresponding plasmonic image, calculated numerically using FDTD methods at 950 *nm* above the metal slab and in the far-field regime at 1 *m*. Figure 4(b) focuses on polystyrene (*n*_*p*_ = 1.61) nanoparticles of various sizes (i.e. *r*_*p*_) and Fig. 4(c) on dielectric nanoparticles of radius *r*_*p*_ = 150 *nm* and varying refractive indices. One can easily see that *σ*_*scat*_ of (5) predicts accurately the features of the maximum plasmonic image for different nanoparticles and they are in fact proportional. As expected 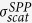 follows closely *σ*_*scat*_, deviating from each other only for large nanoparticles and for larger refractive index values, where there is more diffraction in the radiative channel. The difference seen between the numerical calculations at 950 *nm* and 1 *m* is due to the field dissipation as it propagates in the far-field, which our analytical description and therefore *σ*_*scat*_, 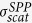 do not account for yet. However, it is interesting to note that when an SPP-induced natural mode is excited (i.e. *r*_*p*_ = 150 *nm* and *n*_*p*_ 3.1), the maximum |*E*|-field intensity of the plasmonic image retains its value. In Fig. 4(a), a map of *σ*_*scat*_ is plotted for loss-less dielectric nanoparticles, where one can identify the plateau of Fig. 4(c) at *n*_*p*_ ~ 2.2 is due to the tail of the second natural mode. In Fig. 5, *σ*_*ext*,*scat*_ is plotted for gold and silver nanoparticles of various sizes. The total extinction cross-section predicts accurately the calculated maximum |*E*|-field of the plasmonic image observed at 950 *nm*, with the radiative component taking significant values even for small metal nanoparticles due to the large refractive index difference between metal and water. It should be noted that the mesh grid for the FDTD calculations was kept constant for all the simulations to ensure that the field enhancement of the excited SPP-wave remained constant and allow quantitative comparison between the plasmonic images calculated of all the nanoparticles. Unfortunately, this also induced inaccuracies to our numerical calculations for the smallest metal nanoparticles due to the stair-case effect in FDTD simulations^31^. This is also clearly evident from Fig. 5, where the maximum |*E*|-field of the plasmonic image fluctuates for small nanoparticles. To overcome this problem, one needs massive computational power and unacceptably long computational time. Nevertheless, the features and patterns are clear and it can be safely concluded that the numerical calculations closely follow our analytical predictions.

### SPP-induced Natural Modes

**Figure 6:**
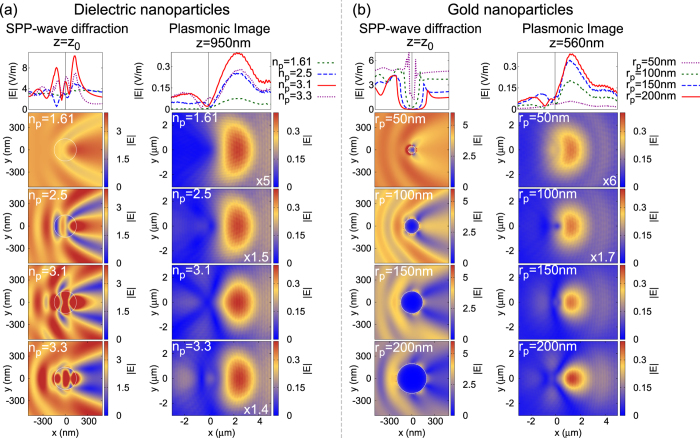
The diffracted fields and plasmonic image for (**a**) various dielectric nanoparticles of *r*_*p*_ = 150 *nm* and (**b**) of gold nanoparticles of different sizes. The left figures of both (**a**,**b**) show the diffracted SPP-wave fields and the right-hand figures their associated plasmonic image. For the *n*_*p*_ = 3.1 and *r*_*p*_ = 150 *nm* nanoparticle, a natural mode is excited.

As it is evident from Figs 3 and 4, SPP-induced natural modes are excited when a SPP-wave interacts with a spherical nanoparticle, more clearly evident for dielectric nanoparticles. Figure 4(c) shows the SPP-induced natural modes have an impact to the plasmonic image’s intensity. Figure 6(a) shows for different dielectric nanoparticles of radius *r*_*p*_ = 150 *nm*, the diffracted fields at a plane parallel to the metal slab passing through the centre of the nanoparticle (left plots) and their corresponding plasmonic image (right plots), calculated with FDTD simulations. For the nanoparticles of refractive indices *n*_*p*_ = 1.61, 2.5 and 3.3, no natural modes are excited as is clearly seen from both the |*E*|-field maps (left plots) and *σ*_*scat*_ in Fig. 4. For a nanoparticle of *n*_*p*_ = 3.1, where *σ*_*scat*_ reaches a local maximum, the displacement current has completed a loop within the nanoparticle, exciting the first SPP-induced natural mode. The top left plot in Fig. 6(a) shows the |*E*|-field inside and immediately around the nanoparticle along the x-axis and passing through the centre of the nanoparticle.

The natural mode excitation produces the largest field enhancement within the nanoparticle. The SPP-induced natural mode excitation (for the nanoparticle of *n*_*p*_ = 3.1) changes the shape of the plasmonic image. It appears more confined and symmetric in shape compared to the plasmonic images away from the natural mode excitation. Also, the amplitude of the plasmonic image fields peak when the natural mode is excited, as *σ*_*scat*_ also predicts.

Figure 6(b) shows a similar study for gold nanoparticles of various radii. Although there is no clear excitation of natural modes, one can clearly see (left figures) the excitation of plasmonic modes on the surface of the nanoparticles. For sub-wavelength nanoparticles, the plasmonic image has a similar shape to the plasmonic image of dielectric nanoparticles. As the size of the nanoparticle increases the plasmonic image becomes more confined and more representative to the geometric characteristics of the nanoparticle, since the SPP-wave can resolve the nanoparticle. It is interesting to note that the plasmonic image shape of a natural mode excitation shows similar features to the image of meso-scopic metal nanoaprticles, and in both cases *σ*_*ext*_ takes similar values. Figures 4, 5, 6 clearly demonstrate that the excitation of natural modes from SPP-waves dramatically alter both the shape and intensity of the plasmonic image. When a natural mode is excited, the plasmonic image is dramatically more bright, while its shape is more confined and symmetric in shape. This is observed for both sub-wavelength and large particles. For biological objects that usually have refractive index of *n*_*p*_ ~ 1.5 that is similar to their aqueous environment *n*_*w*_ = 1.333, natural modes appear for *μm*-sized particles (see Suppl. Mat.). However, since biological objects, such as mitochondria and DNA-molecules, are of ~1.5 *μm* and ~2–3 *μm* in length respectively, natural modes are excited but also strongly dependent on the object’s shape.

Since plasmonic imaging is increasingly used more widely in biological sciences, due to its label-free and real-time nature, as well as electrocatalytic measurements, our findings provide a valuable guide towards interpeting the observed images and obtaining spectroscopic information. The theoretical model presented in this articles formulates the physical dynamics producing the plasmonic image. With the aid of this model, it is shown that the SPP-cross-section is linearly proportional to the maximum |*E*| value of the plasmonic image recorded in the far-field. Most importantly though, natural modes are excited from the SPP-wave, despite their evanescent nature and exponential field decay across the nanoparticle’s diameter. In fact, the natural modes dominate the plasmonic image recorded in the far-field, by altering its intensity and shape. These conclusions open the route towards the real-time characterization of even sub-wavelength nanoparticles with label-free plasmonic imaging techniques.

## Methods

### SPP-Scattering and Extinction Cross-sections and efficiencies

Assuming a putative sphere of radius *r* around the nanoparticle, then the electromagnetic energy crossing its surface *A*^32^ is 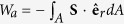, where **S** is the Poynting vector of the wave. Therefore, *W*_*ext*_ = *W*_*scat*_ − *W*_*abs*_, where *W*_*i*_ is the energy rate associated with the scattered, absorbed and extincted incident wave crossing surface *A*, assuming the nanoparticle is embedded in a non-absorbing medium. The Poynting vectors for the incident, scattered and extinction fields are:


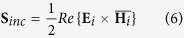



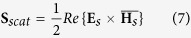



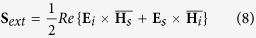


where {} denotes a time averaged vector and 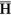 the complex conjugate of **H**. Assuming that the putative sphere has a radius equal to the particle’s (*r* = *r*_*p*_), the scattered and extinction energy rates are given by:


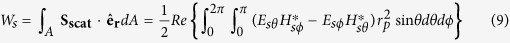



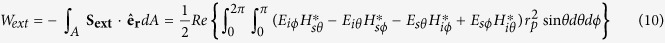


The scattering and extinction cross-sections are given by: *σ*_*scat*,*ext*_ = *W*_*scat*,*ext*_/*I*_*i*_, where *I*_*i*_ is the incident intensity on the nanoparticle, which is obtained by integrating the amplitude of the the incident SPP wave on the nanoparticle:


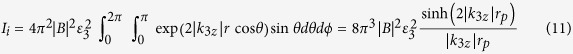


which as expected is a function of the nanoparticle’s radius *r*_*p*_.

Therefore, the scattering and extinction cross-sections are given by:


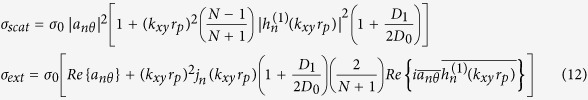


where 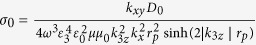, 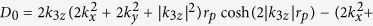 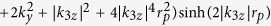 and 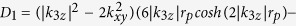 (3 + 4(|*k*_3*z*_|*r*_*p*_)^2^sinh(2|*k*_3*z*_|*r*_*p*_))). Finally, the scattering and extinction efficiencies are given by: 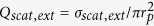


### Finite Difference Time Domain (FDTD) simulations

All numerical field distributions, cross-sections and efficiencies in this manuscript are calculated using the commercial software Lumerical 8.7.3^31^. The electric permittivity of gold is fitted to Johnson and Christy’s experimental data^30^. The total-field scattered technique was used to reduce the needed computing resources. A converging test is performed by starting the simulation with a coarse mesh and reducing the mesh-cell size until consecutive simulations produced closely matched results. A converging test is performed initially for the gold slab, when there is no nanoparticle present, followed by a converging test for the nanoparticle. The final mesh cell for the gold film is 10 *nm* × 10 *nm* × 1 *nm* and for the particle *r*_*p*_/15. The simulation time was set to 1000*fs*, which is about 10 times larger than the time required by the simulation to converge. At the end of each simulation though, I also ensure that all field components have decayed to zero, which means that the simulation has run for sufficiently long time for the Fourier transformation to be valid. The source used was Total Field Scattered Field (TFSF) plane wave source, which separates the computation into two distinct regions: (i) Total Field region inside the source box that includes that sum of the incident and scattered fields and (ii) Scattered Field region outside the source box that includes only the scattered fields^31^. The scattering cross-sections are obtained by integrating the power flowing outwards through a box of monitors surrounding source, and therefore it records only the scattered fields. The absorptions and extinction cross-sections are also calculated, by integrating the net power flowing inwards through a box of monitors surrounding the particle (*i.e.* inside the source box). It should be noted that due to the *μm*-sized characteristics of the plasmonic image from *nm*-sized particles, a very large simulation space was needed to record the plasmonic image and a small mesh to resolve the nanoparticles properly. Hence each simulation needed ~2.5–3 days to converge.

## Additional Information

**How to cite this article**: Demetriadou, A. The impact of natural modes in plasmonic imaging. *Sci. Rep.* **5**, 18247; doi: 10.1038/srep18247 (2015).

## Supplementary Material

Supplementary Information
