# Supplementary material for: The impact of natural modes in plasmonic imaging
Source: Sci Rep. 2015 Dec 16;5:18247. doi: 10.1038/srep18247 (PMC4680891; doi:10.1038/srep18247)
Supplement: Supplementary Information [file srep18247-s1.pdf]

Supporting information for: The impact of natural modes in plasmonic imaging

Angela Demetriadou*

Department of Chemistry, Imperial College London, London, SW7 2AZ, United Kingdom

Department of Physics, Imperial College London, London, SW7 2AZ, United Kingdom

E-mail: a.demetriadou06@imperial.ac.uk

Field equations

The reduced electric field equations for the incident wave on the nanoparticle are given by:

$$\begin{aligned} E_{ir} &= \frac{B\exp\left(|k_{3z}|r\cos\theta\right)}{\omega\varepsilon_3\varepsilon_0}\sum_n^{\infty}4\pi i^n E_{0r} j_n\left(k_{xy}r\right) \\ E_{i\theta} &= \frac{B\exp\left(|k_{3z}|r\cos\theta\right)}{\omega\varepsilon_3\varepsilon_0}\sum_n^{\infty}4\pi i^n E_{0\theta} j_n\left(k_{xy}r\right) \\ E_{i\phi} &= \frac{B\exp\left(|k_{3z}|r\cos\theta\right)}{\omega\varepsilon_3\varepsilon_0}\sum_n^{\infty}4\pi i^n E_{0\phi} j_n\left(k_{xy}r\right) \end{aligned} \quad (S1)$$

*To whom correspondence should be addressed

and for the scattered wave from the nanoparticle:

$$
\begin{aligned}
E_{sr} &= \frac{B \exp\left(|k_{3z}| r \cos\theta\right)}{\omega \varepsilon_3 \varepsilon_0} \sum_{n}^{\infty} 4\pi i^n a_{nr} E_{0r} h_n^{(1)}\left(k_{xy} r\right) \\
E_{s\theta} &= \frac{B \exp\left(|k_{3z}| r \cos\theta\right)}{\omega \varepsilon_3 \varepsilon_0} \sum_{n}^{\infty} 4\pi i^n a_{n\theta} E_{0\theta} h_n^{(1)}\left(k_{xy} r\right) \\
E_{s\phi} &= \frac{B \exp\left(|k_{3z}| r \cos\theta\right)}{\omega \varepsilon_3 \varepsilon_0} \sum_{n}^{\infty} 4\pi i^n a_{n\phi} E_{0\phi} h_n^{(1)}\left(k_{xy} r\right)
\end{aligned} \tag{S2}
$$

where:

$$
\begin{aligned}
E_{0r} &= -i|k_{3z}| \sin\theta \cos\phi - i\frac{k_y |k_{3z}|}{k_x} \sin\theta \sin\phi - \left(k_x + \frac{k_y^2}{k_x}\right) \cos\theta \\
E_{0\theta} &= -i|k_{3z}| \cos\theta \cos\phi - i\frac{k_y |k_{3z}|}{k_x} \cos\theta \sin\phi + \left(k_x + \frac{k_y^2}{k_x}\right) \sin\theta \\
E_{0\phi} &= i|k_{3z}| \sin\phi - i\frac{k_y |k_{3z}|}{k_x} \cos\phi
\end{aligned} \tag{S3}
$$

From the equations above, one can obtain the tangential magnetic field equations through Maxwell's equation $\nabla \times E = -\frac{\partial B}{\partial t}$, leading to:

$$
\begin{aligned}
H_{i\theta}^* &= -\frac{H_0^*}{r} \frac{B^* \exp\left(|k_{3z}| r \cos\theta\right)}{\omega \varepsilon_3 \varepsilon_0} 4\pi \left(-i^n\right) E_{0\phi}^* \left(|k_{3z}| r \cos\theta j_n^*\left(k_{xy} r\right) + k_{xy} r \left[j_n\left(k_{xy} r\right)\right]'^*\right) \\
H_{i\phi}^* &= \frac{H_0^*}{r} \frac{B^* \exp\left(|k_{3z}| r \cos\theta\right)}{\omega \varepsilon_3 \varepsilon_0} 4\pi \left(-i^n\right) E_{0\theta}^* \Bigg(|k_{3z}| r \cos\theta j_n^*\left(k_{xy} r\right) + k_{xy} r \left[j_n\left(k_{xy} r\right)\right]'^* + |k_{3z}| r \sin\theta j_n^*\left(k_{xy} r\right)
\end{aligned}
$$

$$
\begin{aligned}
H_{s\theta}^* = \frac{H_0^*}{r} \quad & \frac{B^* \exp\left(|k_{3z}| r \cos\theta\right)}{\omega \varepsilon_3 \varepsilon_0} 4\pi(-i^n) E_{0\phi}^* \Big[a_{nr}^* h_n^{(1)*}\left(k_{xy} r\right) - \\
& a_{n\phi}^* \left(h_n^{(1)*}\left(k_{xy} r\right)\left(1 + |k_{3z}| r \cos\theta\right) + r k_{xy} \left[h_n^{(1)}\left(k_{xy} r\right)\right]'^*\right)\Big] && \text{(S5)} \\
H_{s\phi}^* = \frac{H_0^*}{r} \quad & \frac{B^* \exp\left(|k_{3z}| r \cos\theta\right)}{\omega \varepsilon_3 \varepsilon_0} 4\pi(-i^n) E_{0\theta}^* \Big[a_{n\theta}^* \left(h_n^{(1)*}\left(k_{xy} r\right)\left(1 + r|k_{3z}| \cos\theta\right) + r k_{xy} \left[h_n^{(1)}\left(k_{xy} r\right)\right]'^*\right) - \\
& -a_{nr}^* h_n^{(1)*}\left(k_{xy} r\right) \left(1 - |k_{3z}| r \sin\theta \frac{E_{0r}^*}{E_{0\theta}^*}\right)\Big] && \text{(S6)}
\end{aligned}
$$

where * denotes a complex conjugate.

Derivation of the scattering and extinction cross-sections

If we assume a putative sphere of radius r enclosing the nanoparticle, then the electromagnetic energy crossing this sphere's surface A is given by[?] $W_i = -\int_A \mathbf{S}\cdot\hat{\mathbf{e}}_r dA$, where $\mathbf{S}$ is the Poynting vector of the wave. Therefore, $W_{ext} = W_{scat} + W_{abs}$, where W_{ext}, W_{scat} and W_{abs} are the energy rates associated with the extincted, scattered and absorbed waves. The Poynting vectors are given by:

$$\mathbf{S}_i = \frac{1}{2}Re\{\mathbf{E}_i \times \mathbf{H}_i^*\} \tag{S7}$$

$$\mathbf{S}_{scat} = \frac{1}{2}Re\{\mathbf{E}_s \times \mathbf{H}_s^*\} \tag{S8}$$

$$\mathbf{S}_{ext} = \frac{1}{2}Re\{\mathbf{E}_i \times \mathbf{H}_s^* + \mathbf{E}_s \times \mathbf{H}_i^*\} \tag{S9}$$

where $\{\}$ denotes a time averaged vector. Therefore, the scattered and extinction energy rates are given by:

$$W_{scat} = \int_A \mathbf{S_{scat}}\cdot\hat{\mathbf{e}}_\mathbf{r} dA = \frac{1}{2}Re\left\{\int_0^{2\pi}\int_0^{\pi}\left(E_{s\theta}H_{s\phi}^* - E_{s\phi}H_{s\theta}^*\right) r^2\sin\theta d\theta d\phi\right\} \tag{S10}$$

$$W_{ext} = -\int_A \mathbf{S_{ext}}\cdot\hat{\mathbf{e}}_\mathbf{r} dA = -\frac{1}{2}Re\left\{\int_0^{2\pi}\int_0^{\pi}\left(E_{i\phi}H_{s\theta}^* - E_{i\theta}H_{s\phi}^* - E_{s\theta}H_{i\phi}^* + E_{s\phi}H_{i\theta}^*\right) r^2\sin\theta d\theta d\phi\right\} \tag{S11}$$

Scattering Cross-section

The terms inside the integral for W_{scat} give:

$$\begin{aligned} E_{s\theta}H_{s\phi}^* - E_{s\phi}H_{s\theta}^* = & \frac{|B|^2 H_0^* \exp\left(2|k_{3z}|r\cos\theta\right)}{w^2\varepsilon_3^2\varepsilon_0^2 r}16\pi^2(-i^{2n})\left[\left(|E_{0\theta}|^2 + |E_{0\phi}|^2\right)\times\right. \\ & \times\left[|a_{n\theta}|^2\left(|h_n^{(1)}(k_{xy}r)|^2(1+|k_{3z}|r\cos\theta) + rk_{xy}h_n^{(1)}(k_{xy}r)\left[h_n^{(1)}(k_{xy}r)\right]'^*\right) - \right. \\ & \left. \left. -a_{n\theta}a_{nr}^*|h_n^{(1)}(k_{xy}r)|^2\right] + a_{n\theta}a_{nr}^*|h_n^{(1)}(k_{xy}r)|^2|k_{3z}|r\sin\theta E_{0\theta}E_{or}^*\right] \end{aligned} \tag{S12}$$

The SPP-component of the above equation is the first term in the square parenthesis that is multiple of $(|E_{0\theta}|^2 + |E_{0\phi}|^2)$, and the radiative components multiple of a_{nr}. Integrating over the SPP component leads to:

$$\begin{aligned} W_s^{SPP} &= \frac{4\pi^3 k_{xy}|B|^2 D_0}{w^3 \varepsilon_3^2 \varepsilon_0^2 \mu\mu_0 k_{3z}^3 k_x^2 r^3} |a_{n\theta}|^2 \\ &= W_0 |a_{n\theta}|^2 \end{aligned} \quad \text{(S13)}$$

where $W_0 = \frac{4\pi^3 k_{xy}|B|^2 D_0}{w^3 \varepsilon_3^2 \varepsilon_0^2 \mu\mu_0 k_{3z}^3 k_x^2 r^3}$, taking into account that $H_0 = \frac{1}{i\omega\mu\mu_0}$, $H_0^* = \frac{i}{\omega\mu\mu_0}$, $-i^{2n} = 1$, $i|h_n^{(1)}(k_{xy}r)|^2$ is imaginary, the Wronskian:

$$Re\left\{ih_n(k_{xy}r)\left[h_n^{(1)}(k_{xy}r)\right]^{'*}\right\} = j_n y_n' - j_n' y_n = \frac{1}{(k_{xy}r)^2} \quad \text{(S14)}$$

and the integral:

$$\int_0^{2\pi}\int_0^{\pi}\left(|E_{0\theta}|^2 + |E_{0\phi}|^2\right)\exp(2|k_{3z}|r\cos\theta)\sin\theta d\theta d\phi = \frac{k_{xy}^2 \pi D_0}{2k_{3z}^3 k_x^2 r^3} \quad \text{(S15)}$$

where $D_0 = 2k_{3z}r\left(k_{3z}^2 + 2k_{xy}^2\right)\cosh(2k_{3z}r) - \left(k_{3z}^2 + 2k_{xy}^2 + 4k_{3z}^4 r^2\right)\sinh(2k_{3z}r)$.

From equation S12, the radiative contribution to W_{scat} is:

$$W_{scat}^{rad} = W_0 |a_{n\theta}|^2 (k_{xy}r)^2 \left(\frac{N-1}{N+1}\right)|h_n^{(1)}(k_{xy}r)|^2\left(1 + \frac{D_1}{2D_0}\right) \quad \text{(S16)}$$

by taking into account that:

$$\int_0^{2\pi}\int_0^{\pi}\exp(2|k_{3z}|r\cos\theta) E_{0\theta}E_{0r}^* \sin^2\theta d\theta d\phi = -\frac{k_{xy}^2 \pi D_1}{4k_{3z}^4 k_x^2 r^4} \quad \text{(S17)}$$

where $D_1 = (6k_{3z}r\cosh(2k_{3z}r) - (3 + 4k_{3z}^2 r^2)\sinh(2k_{3z}r))\left(k_{3z}^2 - 2k_{xy}^2\right)$.

Assuming that the putative sphere around the nanoparticle has a radius $r = r_p$, then the

total scattering cross-section is given by:

$$\boxed{\sigma_{scat} = \sigma_0 |a_{n\theta}|^2 \left[1 + (k_{xy} r_p)^2 \left(\frac{N-1}{N+1}\right) |h_n^{(1)}(k_{xy} r_p)|^2 \left(1 + \frac{D1}{2D_0}\right)\right]} \quad \text{(S18)}$$

where $\sigma_0 = \frac{k_{xy} D_0}{4w^3 \varepsilon_3^4 \varepsilon_0^2 \mu\mu_0 k_{3z}^2 k_x^2 r_p^2 \sinh(2|k_{3z}|r_p)}$.

Extinction cross-section

Following a similar methodology one can derive the extinction cross-section through:

$$W_{ext} = -\frac{1}{2} Re \left\{ \int_0^{2\pi} \int_0^{\pi} \left(E_{i\phi} H_{s\theta}^* - E_{i\theta} H_{s\phi}^* - E_{s\theta} H_{i\phi}^* + E_{s\phi} H_{i\theta}^* \right) r^2 \sin\theta d\theta d\phi \right\} \quad \text{(S19)}$$

The term in the parenthesis is given by:

$$E_{i\phi} H_{s\theta}^* - E_{i\theta} H_{s\phi}^* - E_{s\theta} H_{i\phi}^* + E_{s\phi} H_{i\theta}^* = i \frac{16\pi^2 |B|^2 (-i^{2n}) \exp(2|k_{3z}| r\cos\theta)}{r\omega^3 \varepsilon_3^2 \varepsilon_0^2 \mu\mu_0} \left[\left(|E_{0\theta}|^2 + |E_{0\phi}|^2\right) \times \right.$$
$$\times \left[a_{nr}^* j_n(k_{xy} r) h_n^{(1)*}(k_{xy} r) - a_{n\phi}^* j_n(k_{xy} r) h_n^{(1)*}(k_{xy} r) (1 + |k_3 z| r\cos\theta) \right.$$
$$\left. - a_{n\theta}^* k_{xy} r j_n(k_{xy} r) \left[h_n^{(1)}(k_{xy} r)\right]'^* - |k_{3z}| r a_{n\theta} \cos\theta j_n(k_{xy} r) h_n^{(1)}(k_{xy} r) - k_{xy} r a_{n\theta} h_n^{(1)}(k_{xy} r) \left[j_n(k_{xy} r)\right]' \right] -$$
$$\left. - E_{0\theta} E_{0r}^* |k_{3z}| r \sin\theta \left(a_{nr}^* j_n(k_{xy} r) h_n^{(1)*}(k_{xy} r) + a_{n\theta} j_n(k_{xy} r) h_n^{(1)}(k_{xy} r) \right) \right] \quad \text{(S20)}$$

where again the SPP-component is a multiple of $(|E_{0\theta}|^2 + |E_{0\phi}|^2)$ and the remaining terms contribute to the radiative component for W_{ext} and leads to:

$$W_{ext} = W_0 \left[Re\{a_{n\theta}\} + (k_{xy} r)^2 j_n(k_{xy} r) \left(1 + \frac{D_1}{2D_0}\right) \left(\frac{N-1}{N+1}\right) Re\left\{ i a_{n\theta} h_n^{(1)}(k_{xy} r) \right\} \right] \quad \text{(S21)}$$

by also taking into account that:

$$\begin{aligned} Re\left\{ i a_{n\theta}^* h_n^{(1)*} \right\} &= (Re\{a_{n\theta}\} y_n + Im\{a_{n\theta}\} j_n) \\ Re\left\{ i a_{n\theta} h_n^{(1)} \right\} &= -(Re\{a_{n\theta}\} y_n + Im\{a_{n\theta}\} j_n) = -Re\left\{ i a_{n\theta}^* h_n^{(1)*} \right\} \\ Re\left\{ i a_{n\theta}^* \left[h_n^{(1)}(k_{xy} r)\right]'^* \right\} &= \left(Re\{a_{n\theta}\} y_n' + Im\{a_{n\theta}\} j_n' \right) = -Re\left\{ i a_{n\theta} \left[h_n^{(1)}(k_{xy} r)\right]' \right\} \end{aligned} \quad \text{(S22)}$$

Hence the total extinction cross-section is given by:

$$\boxed{\sigma_{ext} = \sigma_0 \left[Re\left\{a_{n\theta}\right\} + \left(k_{xy}r_p\right)^2 j_n\left(k_{xy}r_p\right)\left(1+\frac{D_1}{2D_0}\right)\left(\frac{N-1}{N+1}\right) Re\left\{ i\overline{a_{n\theta}}\overline{h_n^{(1)}\left(k_{xy}r_p\right)}\right\}\right]} \tag{S23}$$

Biological nanoparticles

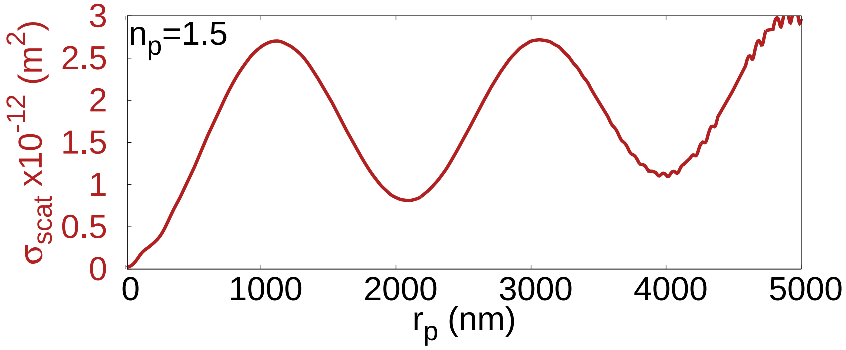

Figure S1: Scattering cross-section for m-nanoparticles of np=1.5 (similar to the refractive index of biological objects) in water (nw=1.333)

In the main manuscript it is demonstrated that natural modes are excited that dominate the intensity of the plasmonic image. However, for nanoparticles with radius $150nm$ (shown in the main manuscript), these modes appear for rather large refractive indices ($n_p = 3.1$), which is a large refractive index for biological particles. In figure S1, the scattering cross-section is plotted for particles with refractive index $n_p = 1.5$ (a realistic value for biological objects) in an aqueous solution. Natural modes are excited for very large particles, due to the small refractive index difference between the nanoparticle and its environment. Since biological objects are usually of μm-dimensions (for example mitochondria are of $\sim 1.5\mu m$ and DNA-molecule $\sim 2-3\mu m$ in length), these natural modes are excited but strongly dependent on the object's shape.
